# An inexpensive point-of-care immunochromatographic test for *Talaromyces marneffei* infection based on the yeast phase specific monoclonal antibody 4D1 and *Galanthus nivalis* agglutinin

**DOI:** 10.1371/journal.pntd.0009058

**Published:** 2021-05-04

**Authors:** Kritsada Pruksaphon, Akarin Intaramat, Pavinee Simsiriwong, Skorn Mongkolsuk, Kavi Ratanabanangkoon, Joshua D. Nosanchuk, Anna Kaltsas, Sirida Youngchim

**Affiliations:** 1 Graduate Program in Microbiology, Faculty of Medicine, Chiang Mai University, Chiang Mai, Thailand; 2 Translational Research Unit, Laboratory of Immunology and Laboratory of Biotechnology, Chulabhorn Research Institute, Bangkok, Thailand; 3 Department of Microbiology, Faculty of Science, Mahidol University, Bangkok, Thailand; 4 Department of Medicine (Infectious Diseases), Albert Einstein College of Medicine, Bronx, New York, United States of America; 5 Department of Medicine, Division of Infectious Diseases, Memorial Sloan Kettering Cancer Center, United States of America; 6 Department of Medicine, Weill Cornell Medical College, Cornell University, New York, United States of America; 7 Department of Microbiology, Faculty of Medicine, Chiang Mai University, Chiang Mai, Thailand; Duke University School of Medicine, UNITED STATES

## Abstract

*Talaromyces marneffei* is a thermally dimorphic fungus that causes opportunistic systemic mycoses in patients with AIDS or other immunodeficiency syndromes. The purpose of this study was to develop an immunochromatographic strip test (ICT) based on a solid phase sandwich format immunoassay for the detection of *T*. *marneffei* antigens in clinical urine specimens. The *T*. *marneffei* yeast phase specific monoclonal antibody 4D1 (MAb4D1) conjugated with colloidal gold nanoparticle was used as a specific signal reporter. *Galanthus nivalis* Agglutinin (GNA) was adsorbed onto nitrocellulose membrane to serve as the test line. Similarly, a control line was created above the test line by immobilization of rabbit anti-mouse IgG. The immobilized GNA served as capturing molecule and as non-immune mediated anti-terminal mannose of *T*. *marneffei* antigenic mannoprotein. The MAb4D1–GNA based ICT showed specific binding activity with yeast phase antigen of *T*. *marneffei*, and it did not react with other common pathogenic fungal antigens. The limit of detection of this ICT for *T*. *marneffei* antigen spiked in normal urine was approximately 0.6 μg/ml. The diagnostic performance of the ICT was validated using 341 urine samples from patents with culture- confirmed *T*. *marneffei* infection and from a control group of healthy individuals and patients with other infections in an endemic area. The ICT exhibited 89.47% sensitivity, 100% specificity, and 97.65% accuracy. Our results demonstrate that the urine-based GNA–MAb4D1 based ICT produces a visual result within 30 minutes and that the test is highly specific for the diagnosis of *T*. *marneffei* infection. The findings validate the deployment of the ICT for clinical use.

## Author summary

Talaromycosis (Penicilliosis marneffei) is a neglected disease that causes an opportunistic systemic mycoses in AIDS and other immune-deficient patients living in Southeast Asia, China and the Indian subcontinent. Although laboratory culture remains a gold standard diagnostic method, it lacks sensitivity and is time-consuming, which results in delayed patient’s treatment and needed care. In this study, we develop an immunochromatographic strip test (ICT) by utilizing a yeast phase specific monoclonal antibody 4D1 and *Galanthus nivalis* agglutinin for detection of *T*. *marneffei* antigens in clinical urine specimens. Our data showed that the assay exhibited high sensitivity (89.47%) and specificity (100%), with its result available within 30 minutes. In addition, this diagnostic assay is inexpensive, reproducible, and simple to perform. Therefore, the *T*. *marneffei* ICT should be considered for clinical application in the context of rapid and affordable point-of-care diagnostic test to reduce the burden of talaromycosis mortality in patients in low resource countries.

## Introduction

The thermally dimorphic fungus *Talaromyces marneffei* (previously *Penicillium marneffei*) causes disseminated mycosis affecting immunocompromised patients living in endemic areas including Southeast Asia, China and the Indian subcontinent [1,2]. In Thailand and Hong Kong, the prevalence of talaromycosis (penicilliosis marneffei) is surpassed only by that of tuberculosis, cryptococcosis and pneumocystis pneumonia [3,4]. Although advanced HIV disease is a well-documented risk factor for talaromycosis, the disease is increasingly reported among patients with other immune defects [5,6].

In the context of clinical laboratory diagnosis, *T*. *marneffei* is usually diagnosed by microscopic identification of the fungus in various clinical specimens and by standard microbiological culture, based on its morphological characteristics and thermally dimorphic properties between 25^o^C (mycelium form) and 37^o^C (yeast form). However, these procedures are relatively time-consuming, requiring about 3-14 days [7,8], which can negatively affect clinical decisions and delay the initiation of appropriate therapy. Thus, a “rapid point of care diagnosis” for *T*. *marneffei* infection is highly desirable, particularly if it could be inexpensive and provide a favorable limit of detection and have high precision and accuracy [9–12]. Ning et al., (2018) systematically reviewed and meta-analyzed the accuracy and diagnostic performance of rapid diagnostics for *T*. *marneffei* (RDTM) previously reported in the literature. They concluded that immunological based methods had high specificity and could potentially be classified for RDTM. However, false-negative or false positive results of these tests were often encountered [13].

Several previous studies have demonstrated that a yeast phase specific monoclonal antibody 4D1 (MAb 4D1; murine IgG1 isotype) is highly specific to the cytoplasmic yeast antigen of *T*. *marneffei*. MAb 4D1 recognizes a 50-180 kDa of N–linked glycoprotein and does not react with other medically important fungal antigens, including the mycelial phase cytoplasmic antigens of *T*. *marneffei* [14–17]. MAb 4D1 has previously been utilized in an inhibition enzyme-linked immunosorbent assay (Inh-ELISA) [15] and in an inhibition lateral flow immunochromatographic strip test (Inh-ICT) [16]; however, the Inh-ICT gave relatively low diagnostic sensitivity and occasional ambiguity in the reading and interpretation of the observed results (personal communication).

In the present study, we developed a lectin–antibody sandwich ELISA using a mannose binding lectin, which recognizes mannose residue called *Galanthus nivalis* agglutinin (GNA) or snowdrop lectin, in conjunction with MAb 4D1. GNA is an agglutinin that is known for its specific binding capacity to α-1,3 terminal mannose residues of oligosaccharides as well as mannoproteins [18,19]. We optimized the conditions for the GNA-MAb 4D1 sandwich ELISA to create a novel sandwich lateral flow immunochromatographic (ICT) strip. The ICT utilizes MAb 4D1 conjugated to 60 nm of gold colloid particle was used as a specific signal generating agent and the carbohydrate binding protein (GNA), immobilized at the test line zone, was used to capture the terminal mannose of *T*. *marneffei* antigen. Urine from talaromycosis patients and controls were used to test the efficacy of the ICT. The diagnostic performances of the novel sandwich ICT were validated in comparison to that of microbiological culture-confirmed talaromycosis marneffei, which is used as the gold standard laboratory diagnosis of *T*. *marneffei* infection.

## Materials and methods

### Ethics statement

The study was approved by the Research Ethics Committee of Faculty of Medicine, Chiang Mai University (approval number MIC-2561-05616/5616), and was carried out in accordance with The Code of Ethics of the World Medical Association (Declaration of Helsinki). All clinical samples investigated in this study were obtained from an existing sample collection. All samples were anonymized.

### Fungal isolates

*T*. *marneffei* ATCC 200051, previously isolated from a bone marrow of AIDS patient at Chiang Mai University Hospital, Chiang Mai, Thailand, was used for all experiments in both the mycelial form and yeast form. The *T*. *marneffei* isolate was maintained by monthly subculture onto Potato Dextrose Agar (PDA; Difco). *T*. *marneffei* was grown on PDA for 7 days at 25°C. Other fungal isolates were obtained and cultivated according to the directions from the American Type Culture Collection (ATCC) or from Department of Medical Services, Ministry of Public Health, Bangkok, Thailand. The fungal strains used this work are summarized in Table 1.

**Table 1:** Fungal isolates.

Fungal species (abbreviation)	Isolate number
*Talaromyces marneffei*	ATCC 200051^**δ**^
*Sporothrix schenckii*	52-S1^**†**^
*Histoplasma capsulatum*	53-H1^**†**^
*Candida albicans*	ATCC 900028^**δ**^
*Candida krusei*	CI
*Candida glabrata*	CI
*Cryptococcus neoformans*	H99^**δ**^
*Aspergillus fumigatus*	55-A1^**†**^
*Penicillium citrinum*	MMC59P12-1^**Ϩ**^
*Penicillium chrysogenum*	ATCC 10106 ^**δ**^
*Pythium insidiosum*	MMC44P21-1^**Ϩ**^
*Pseudallescheria boydii*	MMC60S21-1^**Ϩ**^
*Trichosporon spp*.	CI
*Geotrichum spp*.	CI

**^δ^** Isolate from the American Type Culture Collection, Rockville, MD, USA

**^†^** Isolates from the Institute of Dermatology, Department of Medical Services, Ministry of Public Health, Bangkok, Thailand

**^Ϩ^** Isolate from culture collection in Mycology Unit, Department of Microbiology, Faculty of Medicine, Chiang Mai University, Chiang Mai, Thailand.

CI: Clinical isolates from blood samples of infected patients

### *T*. *marneffei* cytoplasmic yeast antigen (TM CYA) extraction

TM CYA extraction was carried out as described [15,20]. Briefly, 5x10^6^ conidia/ml of *T*. *marneffei* was cultured in brain heart infusion broth (BHI; Difco) and incubated with a shaking incubator at 37°C for 7 days. After killing with 0.02% (w/v) Merthiolate (Sigma) at room temperature for overnight, the yeast cells were harvested by centrifugation at 4,500 rpm for 10 minutes and mechanically broken with 0.5-mm glass Ballotini beads (BioSpec, Inc.) in a homogenizer (BioSpec, Bartlesville, OK, USA). After that, the protease inhibitor cocktails including iodoacetic acid (IAA; Sigma), phenylmethanesulfonyl fluoride (PMSF; Sigma), and EDTA were added to the homogenizer chamber to give final concentrations of 10 μM, 0.1 mM, and 1 mM, respectively. The homogenate was then centrifuged at 10,000 rpm for 30 minutes at 4°C and the cytoplasmic antigen solution was decanted from the mixture supernatant. The cytoplasmic antigens in yeast or mycelial phase of other human pathogenic fungi were also prepared following this standardized procedure. The fungal protein concentrations of the individual preparations were determined using the Coomassie Brilliant Blue G-250 binding method (Bio-Rad Labs, Hercules, CA, USA) [21].

### Purification of monoclonal antibody 4D1 (MAb4D1)

The murine-derived hybridoma cell line clone 4D1, IgG1 isotype, was maintained in serum free medium (Gibco), and purified by HiTrap column protein G affinity chromatography (GE Healthcare) as described [16]. The purity of the IgG fraction was validated by 10% SDS-PAGE. The concentrations of purified MAb 4D1 were determined by molar extinction coefficient at 280 nm of purified IgG (1.36 for a solution of 1 mg/ml) [22]. The immunoreactivity of MAb 4D1 was confirmed using indirect ELISA and Western blotting [15].

### Standardization of GNA-MAb 4D1 sandwich ELISA for detection of TM CYA and other pathogenic fungal antigen

**Fig 1:**
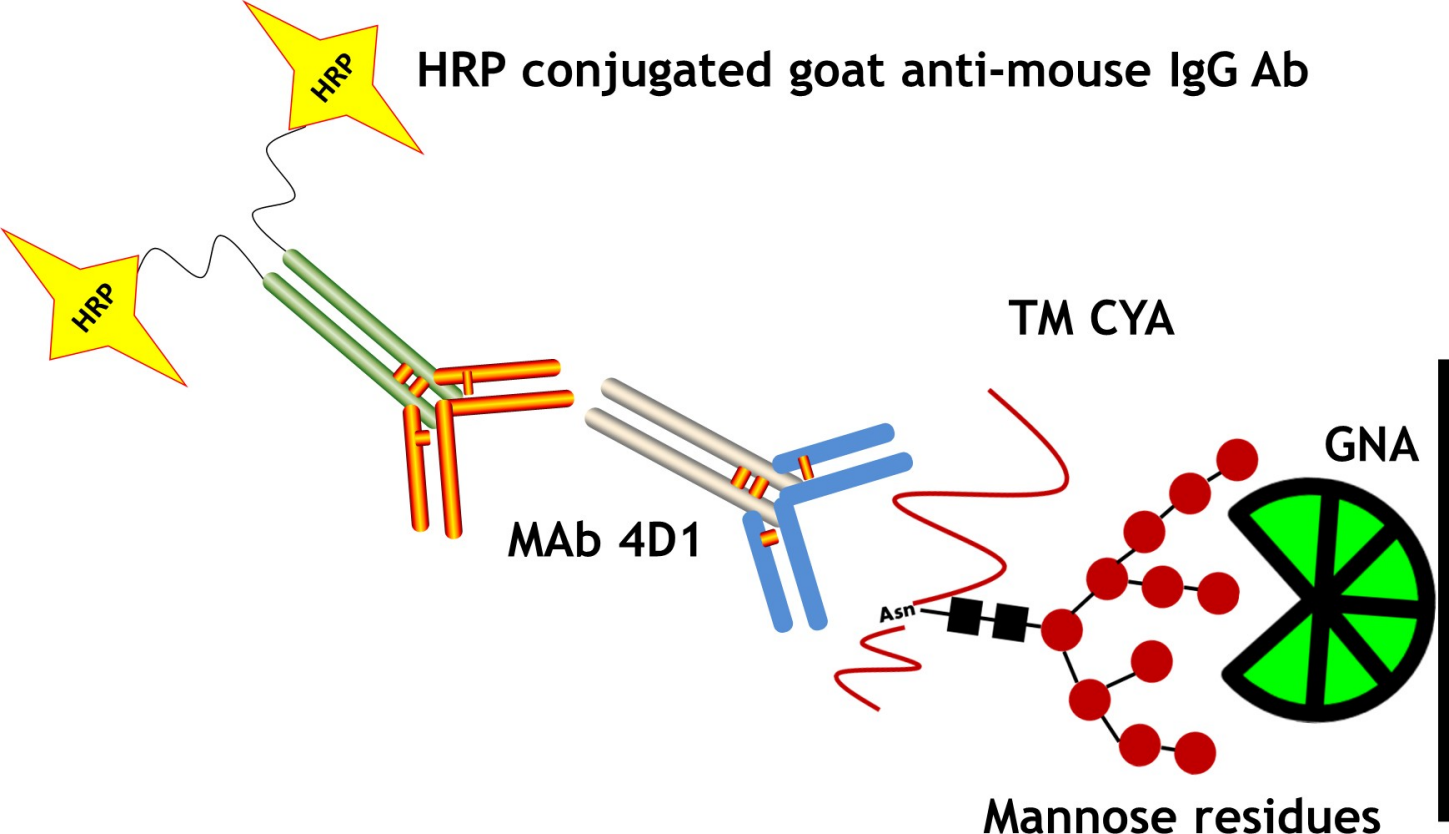
A schematic diagram of interactions between the carbohydrate binding lectin GNA, MAb 4D1 and TM CYA in the sandwich ELISA. The yeast phase-specific MAb4D1 was employed to recognize the core peptide epitope of TM CYA while the mannose-binding lectin (*Galanthus nivalis* agglutinin, GNA), recognized the terminal mannose residues of TM CYA.

A sandwich ELISA using MAb 4D1 and GNA lectin to capture the terminal mannose residues of TM CYA was utilized to standardize and optimize the detection of TM CYA (Fig 1.) Purified GNA lyophilized powder from *G*. *nivalis* (snowdrop) bulbs (Sigma) was solubilized according to the manufacturer’s instructions.

#### Incubation plate

A 96 well U bottom microplate (Nunc A/S, Kamstrup, Denmark) was treated with 200 μl of 1.5% (w/v) BSA (Sigma) in phosphate buffer saline (PBS) pH 7.2 and incubated at 37°C for 60 minutes. Thereafter, the plate was washed five times with 0.05% Tween 20 in phosphate buffer saline pH 7.2 (PBST). Fifty μl of TM CYA or other pathogenic fungal antigen (at concentrations from 50–0.2 μg/ml) in diluting buffer (0.1% w/v BSA in PBST) was added to the BSA treated wells. Subsequently, an equal volume of MAb 4D1 (1.5 μg/ml) was added to each well and the plate was incubated on a shaker at 25°C for 30 minutes at 250 rpm, and then incubated at 4°C overnight to enable the formation of MAb 4D1–TM CYA immune complex.

#### Reaction plate

Fifty μl of 5 μg/ml purified GNA in 0.06 M carbonate coating buffer pH 9.6 was immobilized to wells of Maxisorp 96-well microtiter plates (Nunc A/S) by incubation overnight at 4°C. Unbound GNA was removed by washing. BSA (1.5% w/v) in PBST was added and the plate was incubated at 37°C for 60 minutes. Then, the plate was washed five times with PBST. Aliquots (50 μl) of the contents of each well in the incubation plate (containing a mixture of MAb4D1–TM CYA or other pathogenic fungal antigen) were transferred to respective wells in the reaction plate and allowed to react with the immobilized GNA for 60 minutes at 25°C. The plate was washed again and HRP conjugated goat anti-mouse IgG antibody (Jackson, West Grove, PA, USA) diluted 1:10,000 in diluting buffer was added to each well followed by 60 minute incubation. After washing, H_2_O_2_/TMB substrate (BioFX Laboratories, SurModics IVD, Eden Prairie, MN, USA) was added and the enzymatic reaction was allowed to take place for 20 minutes at room temperature in the dark. The reaction was terminated by adding 2N H_2_SO_4_. The OD450 was measured against a reference at OD570 on an ELISA plate reader (Shimadzu model UV-2401PC, Kyoto, Japan). The test was performed in triplicates.

### Principle and generation of the GNA-MAb 4D1 sandwich ICT strip for the detection of TM CYA

#### (I) Principle of assay format

**Fig 2:**
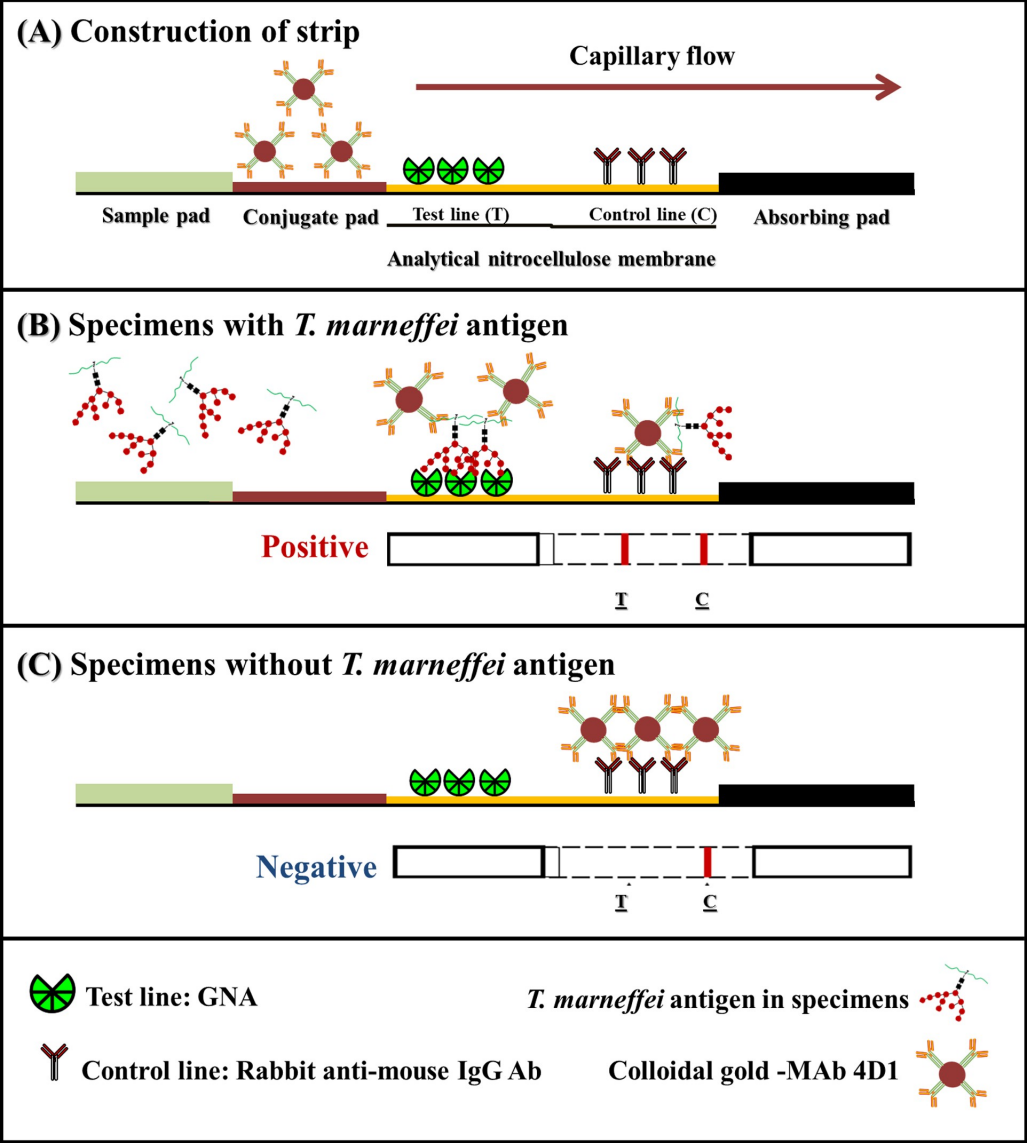
The cartoon formats of GNA–MAb 4D1 sandwich ICT strip for rapid detection of *T*. *marneffei* antigen from a clinical sample. (A), the schematic presentation of the positions where MAb 4D1, GNA and rabbit anti-mouse IgG antibodies are immobilized in an analytical nitrocellulose membrane. (B), the reactions that occur on the MAb 4D1 – GNA strip in the presence of *T*. *marneffei* antigen and (C), in the absence of *T*. *marneffei* antigens. The red–purple color of colloidal gold conjugate appears at the test line and/or control line, depending on the presence of *T*. *marneffei* in the clinical sample.

The sandwich ICT format was applied to detect *T*. *marneffei* antigens in urine samples. MAb 4D1–colloidal gold conjugate was used to generate an assay signal. GNA was immobilized on the test line and rabbit anti-mouse IgG was immobilized on the control line of the micro-porous analytical nitrocellulose membrane. The ICT starts with an absorbing pad that enables the specimen to move along the strip via capillary tension to rehydrate the MAb 4D1–CG conjugate, which then will flow across the sample application pad. When *T*. *marneffei* antigen is present in the specimen, *T*. *marneffei* antigen will bind to the MAb 4D1–CG conjugate and will be captured by the GNA immobilized at the test line (T). This establishes a colored test line and represents a positive result. If the *T*. *marneffei* antigen is absent or below the cut-off point (lower than the limit of detection), there is no visible colored at the test line (T), and this represents a negative result. As an internal control, immobilized rabbit anti mouse IgG reacts with the MAb 4D1-CG conjugate to form a control line (C), indicating that the assay has been performed properly (Fig 2).

#### (II) Preparation of colloidal gold nanoparticle - MAb 4D1 signal generator

MAb 4D1-colloidal gold (MAb 4D1–CG conjugate) is the signal generator used for the detection of *T*. *marneffei* antigens in clinical samples. MAb 4D1 was conjugated to CG particle (60 nm in diameter) by isoelectric point dependent passive adsorption [23]. An aliquot (0.6 ml) of CG suspension (Arista, Allentown, PA, USA) was adjusted to pH 9.0 with 0.1 M Na_2_CO_3_. Purified MAb 4D1 in deionized water (final concentration 7 μg/ml) was added to each well of the CG suspension and gently mixed for 60 minutes at room temperature. The unoccupied surface of the CG particle was blocked with 60 μl of 5% (w/v) casein (Sigma) dissolved in 2 mM Na_2_B_7_O_4_ pH 9.0 for 15 minutes. The MAb 4D1–CG conjugate was centrifuged at 10,000 rpm at 4^o^C for 15 min and the supernatant was discarded. A suspension buffer of MAb 4D1–CG conjugate [0.5% (w/v) of casein, 20% (w/v) of sucrose (Sigma) dissolved in 2 mM Na_2_B_7_O_4_ pH 9.0] was added, and the MAb 4D1–CG conjugates were centrifuged again under the same procedures. After removing the supernatant, the final volume of each aliquot was adjusted to 0.1 ml with MAb 4D1–CG conjugate suspension buffer. To produce the conjugate releasing pads, MAb 4D1–CG conjugate suspensions (5 μl) were individually applied onto a 0.4 x 0.5 cm glass fiber piece (Whatman Schleicher & Schuell, Dassel, Germany) and then heated in a 37°C incubator for 60 minutes. The conjugate releasing pads were further dried in a 30% dehumidifier cabinet overnight before being assembled into the ICT strip system.

#### (III) Establishment of test line and control line onto analytical nitrocellulose membrane

Immobilizations of the GNA and the internal isotype antibody control (rabbit anti-mouse IgG, Sigma) onto the analytical nitrocellulose membranes were established by the physical adsorption in their respective line patterns using a BioDot17x100TM (BioDot, Irvine, CA, USA) dispensing platform. The rate of injection was adjusted to 1 μl/cm. The nitrocellulose membrane (AE99; Whatman Schleicher & Schuell) was sprayed at the test line with purified GNA (10 mg/ml diafiltrated in 10 mM sodium phosphate buffer containing 10 mM sodium chloride pH 7.0) and rabbit anti-mouse IgG (0.5 mg/ml in 10 mM sodium phosphate buffer pH 7.0) was dispensed to the control line. The immobilized nitrocellulose membrane was immediately dried in 30% dehumidifier cabinet for 60 minutes. Then, the nitrocellulose membrane was blocked by submersion into blocking reagent [0.5% (w/v) of casein, 0.5% (w/v) of sucrose and 0.2% (w/v) of trehalose (Sigma) dissolved in 10 mM sodium phosphate buffer pH 7.4]. The blocked nitrocellulose membranes were then dried in a dehumidifier cabinet for 60 minutes and stored at room temperature for 24 hours before being assembled into the ICT strip system.

#### (IV) Preparation of the sample application pad

The sample application pad used in the ICT construction was prepared by pre-treatment of the 903 specimen collection paper (Whatman Schleicher & Schuell, Dassel, Germany) with a mixture solution of 2% (v/v) Triton X-100 (Sigma) and 0.05% (w/v) of polyvinylpyrrolidone (Sigma) dissolved in 50 mM Tris-HCl, pH 7.4 and then the pad was dried in a 30% dehumidifier cabinet overnight [24].

#### (V) Assembly and production of individual ICT strip

**Fig 3:**
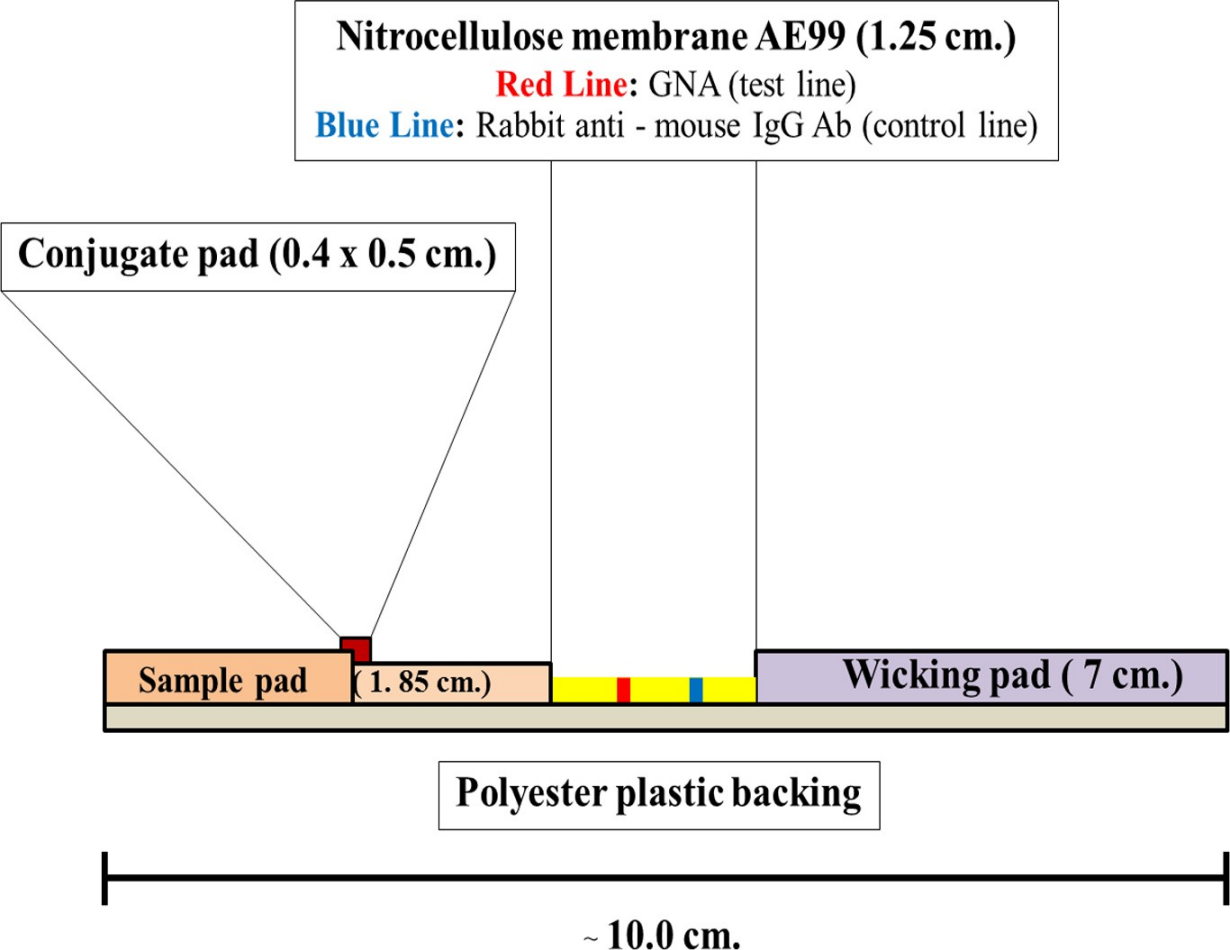
The side view cartoon formats of the components and construction of GNA- MAb 4D1 sandwich ICT strip.

The ICT strip was assembled utilizing 4 major components: (i) the GNA and isotype controlled antibody immobilized nitrocellulose membrane (NC), (ii) the treated sample application pad (SP), (iii) the impregnated conjugate releasing pad (CRP) and (iv) the wicking pad or WP (3 MM chromatography paper, Whatman, Maidstone, England). The components were manually assembled and held permanently in place with a laminating backing card (Self-adhesive polyester backing, Schleicher & Schuell). The assembled cards were then individually cut into 0.4 cm wide and 10 cm long strips using a BioDot1 CM 4000 R guillotine cutter (BioDot, Irvine, CA, USA). The schematic of the ICT strip is detailed in Fig 3.

### Detection of *T*. *marneffei* antigen by the GNA-MAb 4D1 sandwich ICT strip

Urines were aliquoted (150 μl) into a 96-well U shape microtiter plate. ICT strips were dipped vertically with the sample application pad placed into urine specimens. If *T*. *marneffei* antigen was present in the sample, bands formed in both the test line (*T*. *marneffei* antigen, GNA and conjugated MAb 4D1) and the control line. Negative result will show only one band (Rabbit anti-mouse IgG and conjugated MAb 4D1) at the control line. Using direct visualization of bands as readout, the results of the ICT were available within 30 minutes, as determined by independent observations by three experienced laboratory technicians.

### Determination of the lower limit of detection (LOD) and cross reactivity of the GNA-MAb 4D1 sandwich ICT strip

The ICT strip test was used to detect various concentrations of TM CYA in pooled urines from healthy individuals living in an endemic area (Chiang Mai and Bangkok, Thailand). Urines from healthy individuals were spiked with TM CYA to give final TM CYA concentrations of 0.31, 0.62, 1.25, 2.5, 5 and 10 μg/ml. Spiked and non-spiked control urine solutions (150 μl) were pipetted into the microtiter plates. The ICT strips were dipped into the urines, and the visual results were recorded at 30 minutes. The signal intensity of the test lines and control line were also scanned using the Chemidoc-It2, UVP, LLC, Canada and the band intensity was quantified by image J software (version 1.49) [16]. The limit of detection of the test was reported as the lowest concentration of TM CYA that gave a positive signal.

To evaluate the cross reactivity of the ICT, spiked urines from healthy individuals with cytoplasmic proteins (10 μg/ml) derived from different pathogenic fungi were tested. In addition to protein antigens, glucuronoxylomannan (GXM) polysaccharide capsular antigen of *C*. *neoformans* serotype A, strain H99 was purified as described [25] and urine was spiked with this material (10 μg/ml) and tested by the ICT.

### Clinical samples, Healthy urine samples and Sample size calculation

The diagnostic performances of the ICT strip were evaluated in clinical urine. The urine specimens from HIV-seroreactive patients with culture confirmed *T*. *marneffei* infections were collected between September 2004 to September 2019 (n =76) at the clinical pathology unit, Chiang Mai University Hospital, Chiang Mai, Thailand. The urine samples were obtained at the time of diagnosis and stored at -80°C until they were thawed immediately prior to investigation. For the non–*T*. *marneffei* infected control group, urine samples from patients infected with other pathogenic microorganisms (fungus, virus, bacteria and parasites) as well as from healthy individuals living in an endemic area were also selected from our stored urine collection and tested (Table 2).

**Table 2:** Clinical urine samples used in the present study.

Clinical urine samples	Total
Gold standard confirmed of *T*. *marneffei*	76
Other pathogenic microorganism infection (Identified in supplemented Table 1)	184
Normal healthy from endemic area	81
**Total**	**341**

The sample sizes were estimated based on matched case-control studies [26]. The optimized parameter was then determined using the following formula:

$$n=\frac{Z_{1-\frac{\alpha }{2}}^{2}(\hat{P}\hat{Q})}{e^{2}}$$

The minimum acceptable sampling size was 68 cases in both talaromycosis (case) and non–talaromycosis (control) when calculated without the dropout rate. However, regard to the dropout rate was defined as 10%, the minimum acceptable sampling size was 76 cases in both cases and controls.

### Validation of diagnostic performance of the GNA-MAb 4D1 sandwich ICT strip and statistical analysis

Various variables were studied using the number of case-control samples in comparison to the gold standard method, which included the number of true-positive results (TP), the true-negative results (TN), the false-positive results (FP), and the false-negative results (FN). The McNemar’s test for a case-control study was used to calculate the diagnostic sensitivity, specificity and accuracy with their 95% confidence intervals (95% C.I.). Moreover, the consistencies between different tests were determined by Cohen’s kappa coefficient of agreement using GraphPad (QuickCalcs Program, San Diego, CA), which was interpreted according to the kappa statistic values of > 0.81, 0.41 to 0.80, or < 0.40 indicating almost perfect agreement, moderate to substantial agreement, and slight to fair or poor agreement, respectively [27].

## Results

### Detection of TM CYA and other pathogenic fungal antigen by the GNA–MAb 4D1 sandwich ELISA

**Fig 4:**
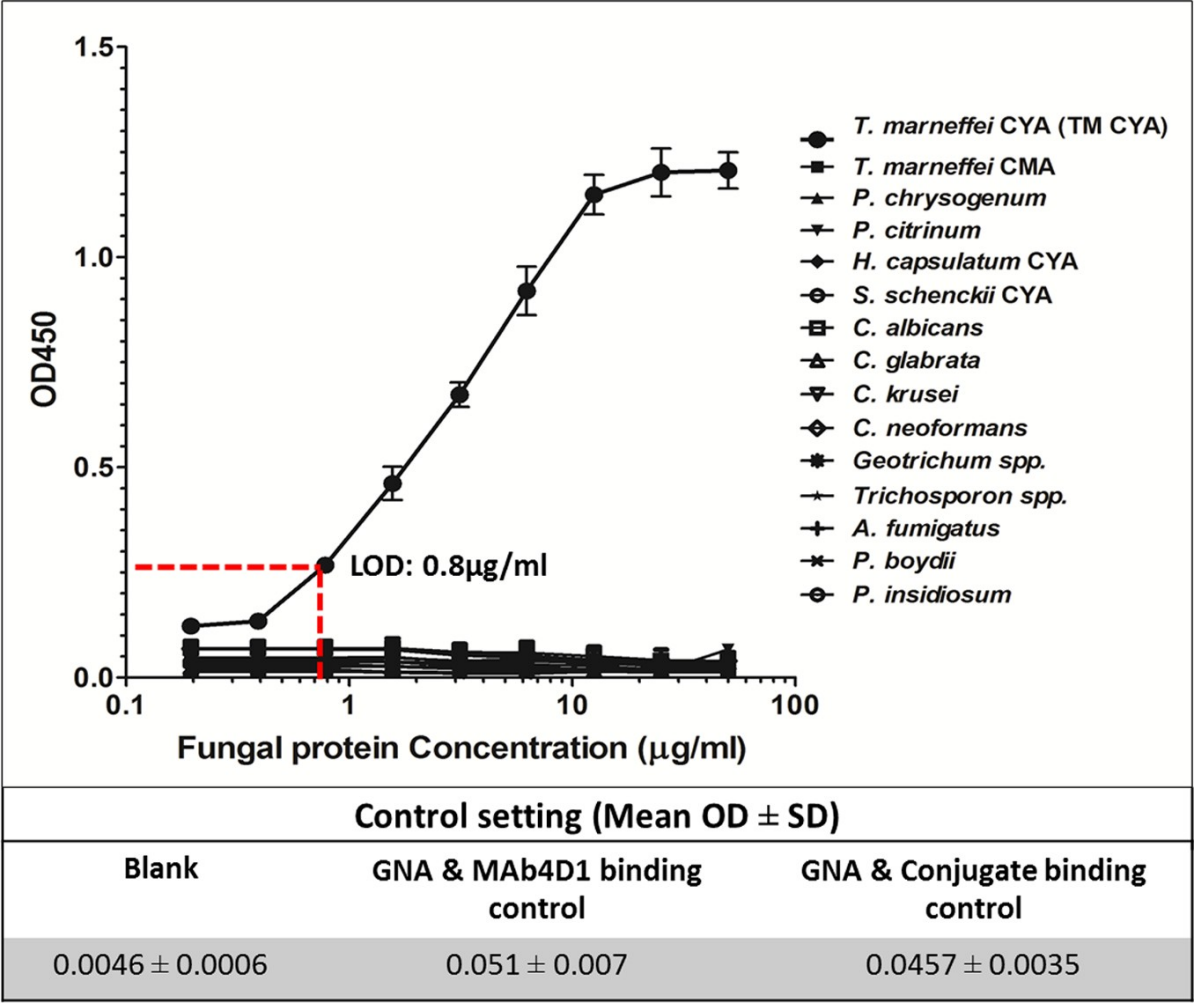
The specificity of the GNA–MAb 4D1 sandwich ELISA in the detection of TM CYA. TM CYA and TM CMA together with 13 other fungal antigen was tested in the ELISA. The positive reaction was indicated by the absorbance measured at OD 450 nm. The fungal protein concentrations were shown in log_10_scale. The results were shown as mean OD ± SD of three independent determinations. Abbreviation: CYA, Cytoplasmic Yeast Antigen; CMA, Cytoplasmic Mycelium Antigen.

The GNA–MAb 4D1 sandwich ELISA format was established to capture the target antigenic mannoprotein in crude extracted of TM CYA (Fig 1). As expected, the bi-functional GNA-MAb 4D1 was selectively reactive against TM CYA, with no detectable reactivity toward other pathogenic fungal antigens as well as *T*. *marneffei* mycelial antigen (TM CMA). For the TM CYA reaction, the limit of detection (LOD) for the standardized sandwich ELISA using GNA–MAb 4D1 was 0.8 μg/ml of TM CYA using an OD 450 nm cut off at 0.250. Moreover, the experimental controls confirmed that GNA alone did not recognize either MAb 4D1 or HRP conjugated goat anti-mouse IgG antibody. Therefore, the positive reaction only occurred when TM CYA was present in the ELISA reaction (Fig 4).

### Development of the GNA-MAb 4D1 sandwich ICT strip for the detection of TM CYA

**Fig 5:**
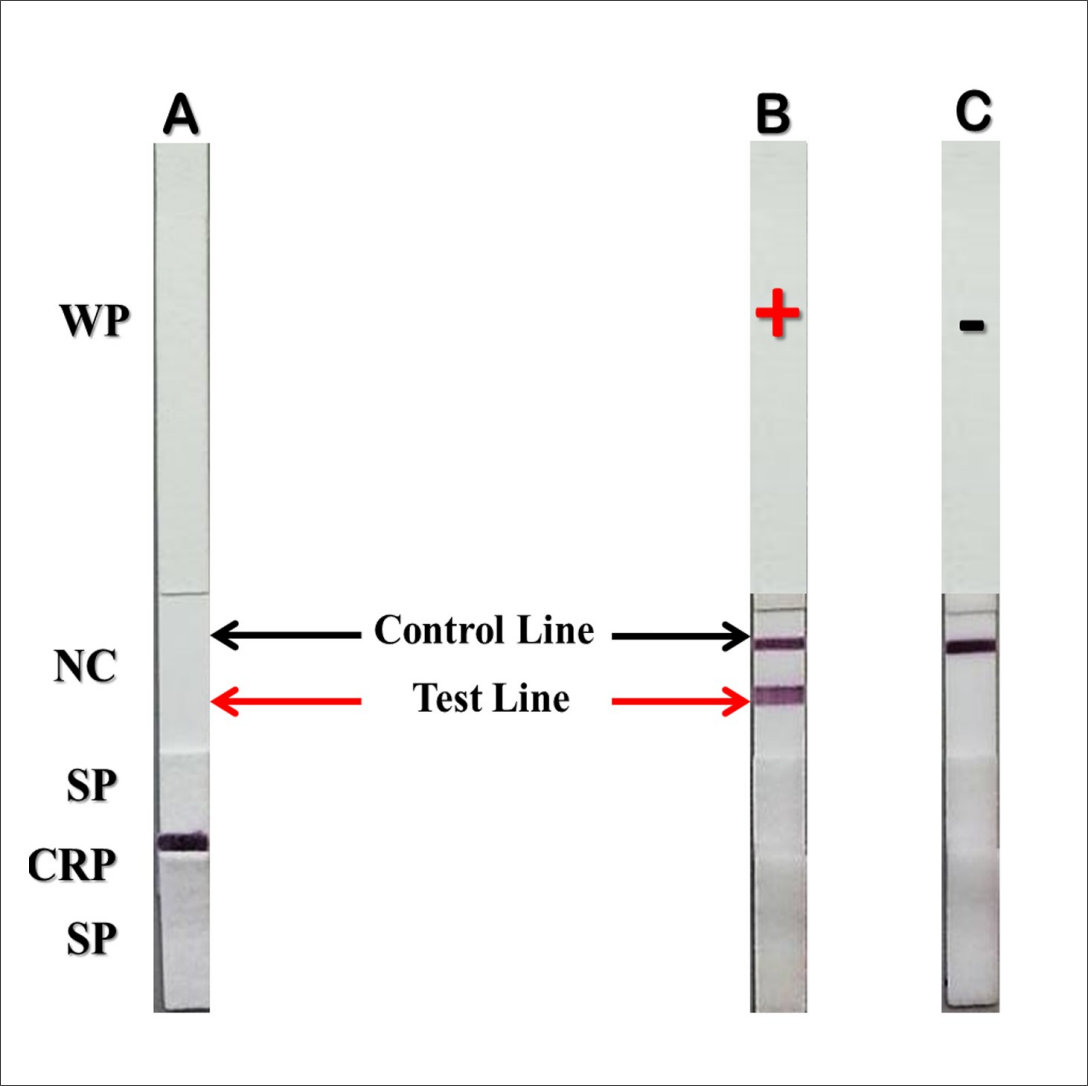
A picture of the GNA-MAb 4D1 sandwich ICT strip in the detection of TM CYA. (A) An untested strip with no signal at the test line or the control line, (B) Positive test result showing intense signals both at the test and at the control lines. (C) Negative result showing signal only at the control line. Abbreviations: WP, wicking pad; NC, analytical nitrocellulose membrane; SP, sample application pad; CRP, conjugate releasing pad.

A sandwich ICT strip for the detection of TM CYA was developed using GNA directly immobilized onto the analytical nitrocellulose membrane with the MAb 4D1-CG conjugate serving as the signal reporter. The specific reaction of TM CYA and MAb 4D1-CG conjugate resulted in the formation of a visible purple immune complex on the test line of the nitrocellulose membrane. The excess MAb 4D1-CG conjugate moved further to the control line which served to verify the validity of the assay (Fig 5).

### Limit of detection (LOD) of the GNA-MAb 4D1 sandwich ICT strip

**Fig 6:**
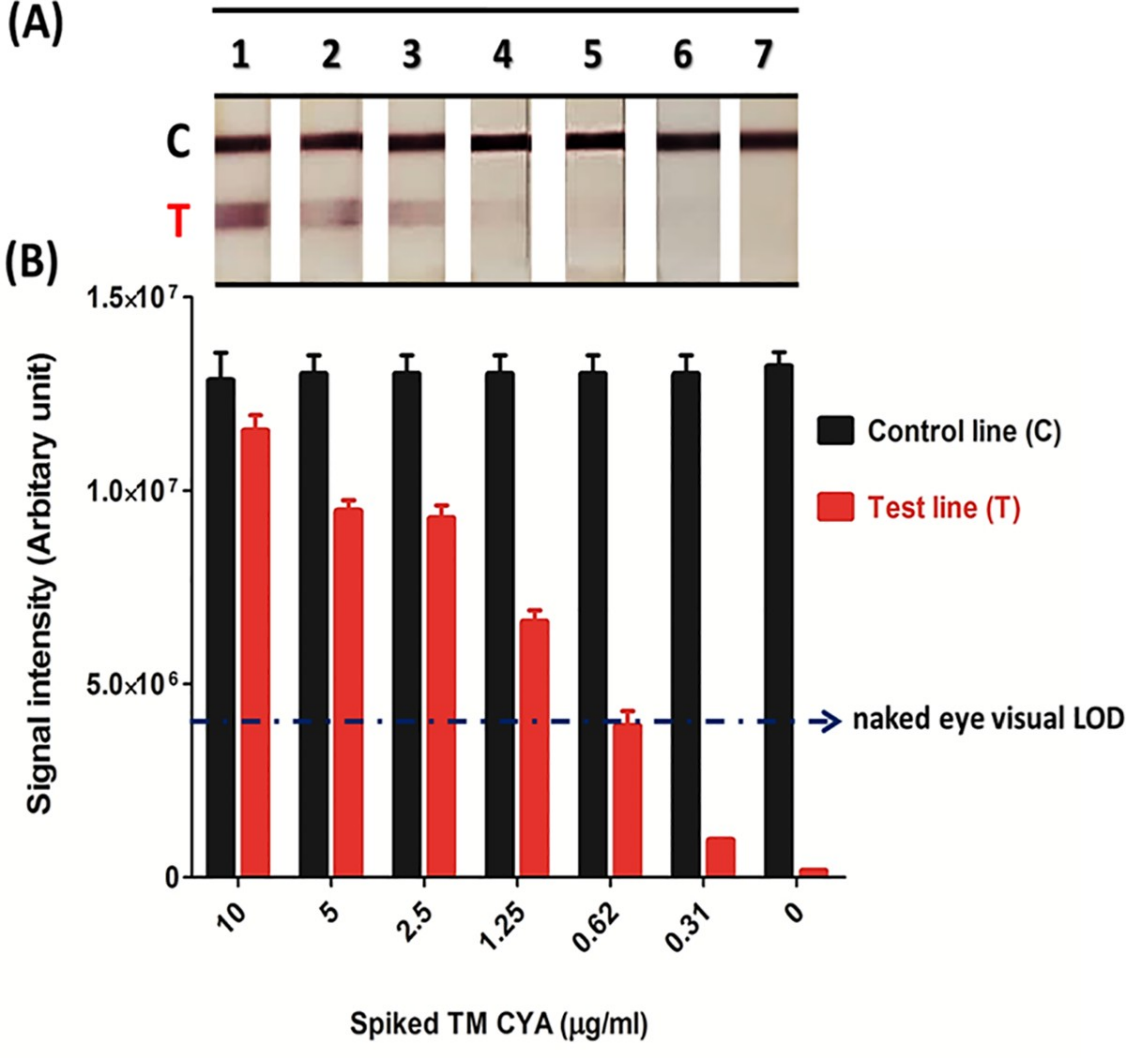
Lower limit of detection (LOD) of the GNA-MAb 4D1 sandwich ICT strip. Normal urine samples from healthy individuals were spiked with TM CYA at the indicated concentrations from 10–0.31 μg/ml. (A) Visual observation, A-G labels represented the concentrations of TM CYA from 10, 5, 2.5, 1.25, 0.62, 0.31, and 0 μg/ml, respectively, which be compatible with TM CYA concentrations that demonstrated in the below graph. (B) Densitometric analysis using UVP vision scanner and image J software. (Abbreviations indicated C: control line, T: test line).

The lower limit of detection of this assay ICT strip was investigated with the urine from normal healthy volunteers spiked with various concentration of TM CYA. Direct visualization found that the LOD for the ICT was 0.62 μg/ml of TM CYA and the readout was achieved within 30 minutes. The LOD of this assay was also quantified and interpreted with the Chemidoc-It2 UVP scanner and image J software (version 1.49). The test line band intensity gradually decreased in a dose-response manner of spiked TM CYA, and the digital methodology produced a similar result to what was achieved with direct visualization (Fig 6).

### Cross reactivity of the GNA-MAb 4D1 sandwich ICT strip

**Fig 7:**
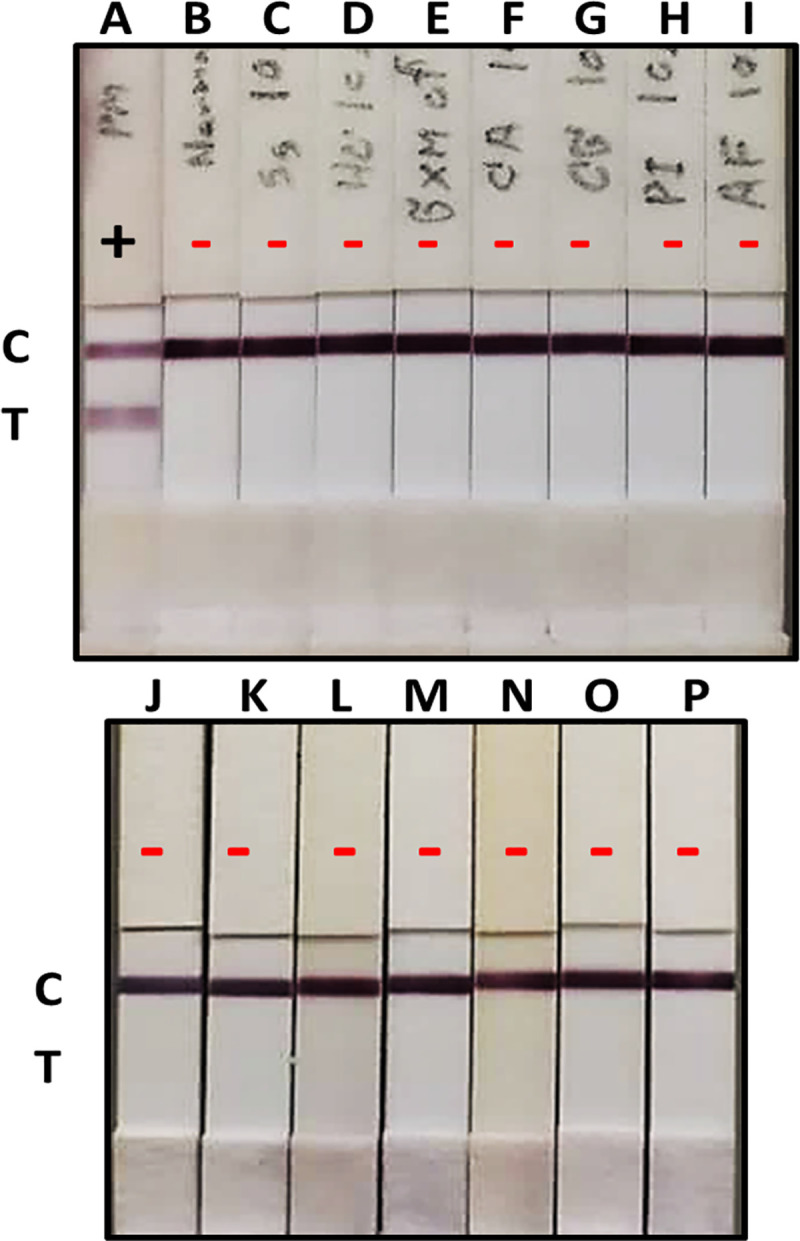
Specificity of the GNA-MAb 4D1 sandwich ICT strip. Normal urine samples from healthy individuals were spiked with various pathogenic fungal antigens at 10 μg/ml. A, TM CYA (served as a positive control); B, normal healthy urine sample (served as a negative control); C, *S*. *schenckii* CYA; D, *H*. *capsulatum* CYA; E, *C*. *neoformans* GXM; F, *C*. *albicans*; G, *C*. *glabrata*; H, *P*. *insidiosum*; I, *A*. *fumigatus*; J, *P*. *citrinum*; K, *P*. *chrysogenum*; L, *P*. *boydii*; M, *C*. *krusei*; N, *Trichosporon* spp.; O, *Geotrichum* spp.; and P, TM CMA. Abbreviations: CYA, Cytoplasmic Yeast Antigen; CMA, Cytoplasmic Mycelium Antigen C, control line; T, test line.

To investigate the specificity of the sandwich ICT, TM CMA as well as crude antigenic proteins extracted from common fungal pathogens (Table 1) were spiked into urine from normal healthy volunteers at a final concentration of 10 μg/ml (equal to the maximum concentration of TM CYA). The sandwich ICT strip was highly specific to TM CYA; the results showed an absence of cross-reactivity, even with the *T*. *marneffei* mycelial extracts, TM CMA (Fig 7). Although this assay gave only qualitative results, these results were agreement with those obtained from GNA-MAb 4D1 sandwich ELISA.

### Detection of *T*. *marneffei* antigenuria by the GNA-MAb 4D1 sandwich ICT strip and diagnostic performance

The diagnostic performance of the sandwich GNA-MAb 4D1 sandwich ICT strip was further conducted using 341 urine samples obtained from patients infected with *T*. *marneffei* or other infections as well as from normal healthy controls. For the 76 confirmed talaromycosis cases, 68 urine samples produced positive ICT results and 8 samples were false negatives. For the control cases, all 265 non–*T*. *marneffei* urine samples were negative by ICT. Among the 265 non–*T*. *marneffei* urine samples, 81 were from normal healthy subjects living in an endemic area (Table 3). The remaining 184 samples were identified as infected with other pathogenic microorganism or they had other underlying diseases associated with immune system impairment (S1 Table).

**Table 3:** Two by Two table analysis of diagnostic performance of GNA-MAb 4D1 sandwich ICT strip.

Sandwich ICT strip results	Gold standard confirmed Talaromycosis		Total
	Positive	Negative	
Positive	68 (TP*)	0 (FP*)	**68**
Negative	8 (FN*)	265 (TN*)	**273**
**Total**	**76**	**265**	**341**

*TP, True Positive; TN, True Negative; FP, False Positive; FN, False Negative

The diagnostic performances of the GNA-MAb 4D1 sandwich ICT was in concordance with the results from culture confirmed talaromycosis. The sandwich ICT strip exhibited a diagnostic sensitivity, specificity and accuracy of 89.47% (95% C.I.; 80.31–95.34), 100% (95% C.I.; 98.62–100) and 97.65% (95% C.I.; 95.43–98.98), respectively. The Cohen’s kappa coefficient analysis was 0.930 (95% C.I.; 0.882–0.978) for the samples assayed by the two different diagnostic techniques, indicating ‘almost perfect agreement’ between the novel sandwich ICT and the gold standard diagnosis of talaromycosis (microbiological culture) [27] (Table 4).

**Table 4:** Summary of diagnostic performance of GNA-MAb 4D1 sandwich ICT strip.

Diagnostic performance criteria	Percentages (95% C.I.)
Sensitivity	89.47 (80.31–95.34)
Specificity	100 (98.62-100)
Accuracy	97.65 (95.43–98.98)
Cohen’s kappa coefficient (κ)	0.930 (0.882-0.978)

### Study on the interference of non–*T*. *marneffei* mannan with the GNA-MAb 4D1 sandwich ICT

**Fig 8:**
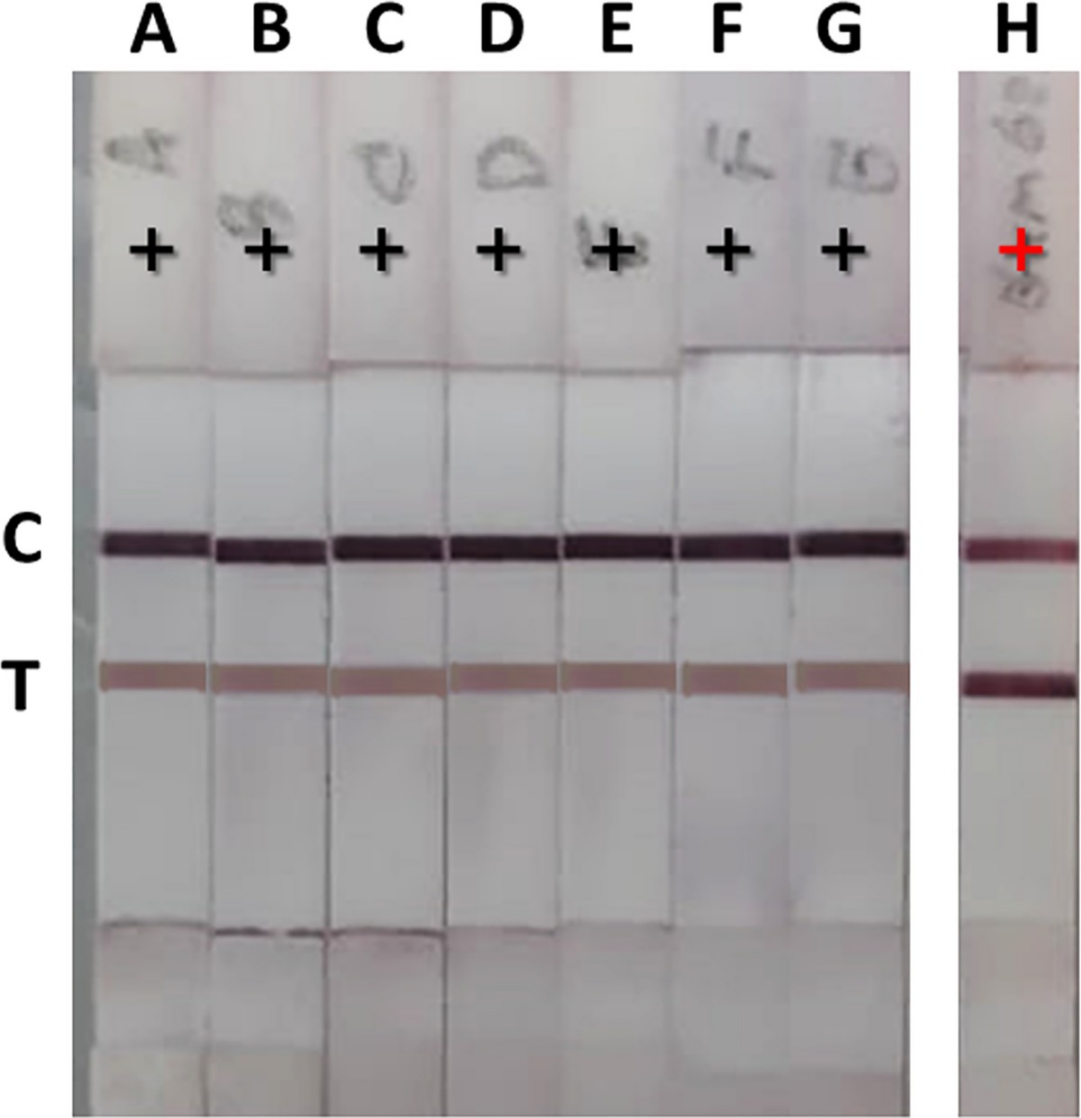
The effect of non–*T*. *marneffei* mannan interference with GNA-MAb 4D1 sandwich ICT strip. *T*. *marneffei* positive urine was spiked with various concentrations of GXM of *C*. *neoformans* (serotype A). A; *T*. *marneffei* positive urine, B–G labels indicated the concentrations of *C*. *neoformans* GXM from 1,000, 500, 250, 125, 62.5, and 31.25 ng/ml, respectively, were spiked in *T*. *marneffei* positive urine. H; to confirm GXM presence in urine, in-house sandwich ICT strip was established for detecting *C*. *neoformans* capsular antigen at the GXM concentration of 31.25 ng/ml.

Since GNA recognizes the terminal mannose residues of fungal antigen, we considered the possibility that mannans released by other fungi in patients co–infected with *T*. *marneffei* could affect the performance of the ICT. For example, infections with both *T*. *marneffei* and *C*. *neoformans* occur in both HIV and non-HIV associated talaromycosis [28–30]. To investigate the possible interference of the major mannan produced by *C*. *neoformans*, the capsular polysaccharide antigen GXM, we spiked *T*. *marneffei* positive urine spiked with different concentrations (1,000–31.25 ng/ml) of purified GXM, as previous studies report that GXM concentrations in the urine of cryptococcosis patients are approximately 170 ng/ml [31]. We found that the presence of GXM did not alter the performance of the ICT (Fig 8). To confirm the presence of GXM in the spiked urine, we performed our in-house ICT strip for *C*. *neoformans* capsular antigen (Fig 8H) [32].

## Discussion

*T*. *marneffei* opportunistically occurs in patients with secondary immunodeficiency syndromes, especially in patients with advanced HIV infection or other diseases associated with cellular immunity impairment [17]. In order to improve the outcomes for patients and to facilitate the earlier administration of appropriate antifungal medications, laboratory diagnosis of talaromycosis must be accurate and rapid. The definitive diagnosis for talaromycosis relies on the direct culture of the fungus from a variety of clinical samples, and it is based on fungal morphology and thermally dimorphism properties [1].This procedure is time-consuming, which may delay the diagnosis of this systemic mycosis, resulting in delays treatment and increases in mortality. Other clinical microscopic diagnostic approaches based on pathological staining (e.g., Hematoxylin and Eosin (H&E), Gomori methenamine silver (GMS) and Periodic acid–Schiff (PAS) staining have been applied to various types of clinical samples including skin scraping, bone marrow and lymph nodes [33]. However, such methods have important limitations since the microscopic morphology of *T*. *marneffei* yeast-like cells are extremely similar to other intracellular pathogen such as *H*. *capsulatum*, *Pneumocystis jirovecii* as well as *Leishmania donovani* [34]. Additionally, tissue processing and these staining techniques can be time intensive.

The rapid diagnostic methods for talaromycosis available to date do not have adequate diagnostic sensitivity, specificity, and accuracy. Although molecular methods e.g., polymerase chain reaction (PCR) based assays, mainly real-time quantitative PCR [35] and nested PCR [36] have been established, the diagnostic sensitivities of PCR based assays are insufficient (range 60-70%). Also, such molecular methods remain impractical for the point of care testing as they are relatively expensive and they require sophisticated instruments and skilled laboratory personnel [12,17]. Therefore, simpler, faster tests with higher sensitivity and specificity are required, and rapid immunological-based methods constitute a rational approach to achieve this goal.

Currently, there is no commercially available rapid immunodiagnostic test for *T*. *marneffei*. There has been interest in utilizing the detection of galactomannans (GM) by the Plateria *Aspergillus* EIA [37,38]; however, significant false positive occur [17], which is not surprising given that GM is produced by a variety of fungi commonly encountered in areas where talaromycosis occurs, including *Aspergillus spp*., *Penicillium spp*., *Paecilomyces spp*., *Purpureocillium licacinum*, *Histoplasma spp*. and *Cryptococcus spp*. [39,40].

We have previously demonstrated the specificity of MAb 4D1 for TM CYA [14–17]. Our prior Inh-ELISA [15] and Inh-ICT [16] have demonstrated the utility of MAb 4D1 in immunodiagnostic strategies.We have now incorporated colloidal gold nanoparticle- conjugated MAb 4D1 and GNA in the development of an inexpensive, user-friendly and efficient ICT for the visual detection of *T*. *marneffei* antigen in urine within 30 minutes. In general, carbohydrate binding proteins or lectins (often called agglutinins) are ubiquitous proteins of non-immune origin. They are extracted from plants, microorganisms, animals; and interact specifically and reversibly with defined monosugars or oligosaccharide structures in accordance with the lock and key model [41,42]. Multiple applications of lectins in the field of biological research, both as reagents and clinical diagnostic tools have been frequently reported [43,44].

For the diagnostic performance of the ICT, sensitivity, specificity, and accuracy of the assay were 89.47%, 100%, and 97.65% respectively. However, since approximately 76% of patients with *T*. *marneffei* infections have positive blood cultures [7,45], it is possible that the sensitivity of this ICT strip might be lower in urine from hemoculture negative talaromycosis due to a lower level of *T*. *marneffei* antigen in urine. However, antigens are frequently detected in the urine by immunodiagnostic methods in histoplasmosis patients without fungemia [46,47]; hence, further testing is required to determine the efficacy of the *T*. *marneffei* ICT in various clinical settings. We unfortunately do not have the clinical information for the 8 false negative cases, which would potentially provide important information on the limitations of the ICT. It is possible that these individuals were either early in their disease process or were already undergoing antifungal drug treatment. The latter is very important as the ICT may be able to guide therapy. As the differential band intensity in Fig 6 is directly associated with the concentration of TM CYA in urine, it may be possible to derive quantitative results using a standardized readout with a biosensor-based machine [48]. Hence, the ICT could define changes in antigen concentration over time, which may correlate with the efficacy of treatment [15,49]. However, the simplicity of interpreting the strips by visual assessment means that the ICT is highly appropriate for utilization in under resourced regions, such as in rural communities, to facilitate an early diagnosis.

It is important to consider the application of the ICT for testing other clinical body fluids. Unfortunately, our ICT strip system cannot be used with serum samples; the physical matrix of serum interferes with the colloidal gold conjugate and leads to the deterioration of colloidal gold and fluid obstruction. When we tested the ICT strip with talaromycosis serum, the diagnostic sensitivity was reduced to 71.43% (15/21). In the future, we may have the opportunity to investigate the efficacy of the ICT system with other body fluids, such as bronchoalveolar lavage or cerebral spinal fluid [50,51].

In conclusion, the GNA-MAb 4D1 sandwich ICT strip is a novel rapid lateral flow diagnostic test that effectively identifies *T*. *marneffei* antigen in clinical urine samples. The assay is inexpensive, reproducible, and extremely easy to perform. As results are available within 30 minutes, the *T*. *marneffei* ICT may facilitate a substantially earlier diagnosis of *T*. *marneffei* infection before the results of other currently applied method are available. Therefore, we recommend that the *T*. *marneffei* ICT strip should be considered for clinical application in the context of rapid diagnosis for talaromycosis.

## Supporting information

S1 Table Urine samples of non–talaromycosis group. (DOCX)
