# Supplementary material for: An inexpensive point-of-care immunochromatographic test for Talaromyces marneffei infection based on the yeast phase specific monoclonal antibody 4D1 and Galanthus nivalis agglutinin
Source: PLoS Negl Trop Dis. 2021 May 4;15(5):e0009058. doi: 10.1371/journal.pntd.0009058 (PMC8096094; doi:10.1371/journal.pntd.0009058)
Supplement: S1 Table — (DOCX) [file pntd.0009058.s001.docx]

**Table S1.** Urine samples of non – talaromycosis group

| **Non - Talaromycosis** | **Total no. of samples** |
| --- | --- |
| *Cryptococcus neoformans* | 14 |
| *Histoplasma capsulatum* | 2 |
| *Candida albicans* | 12 |
| *Candida tropicalis* | 6 |
| *Candida glabata* | 7 |
| *Candida krusei* | 5 |
| *Candida guilliermondii* | 1 |
| *Candida parapsilosis* | 1 |
| Diabetes Mellitus with Candidiasis | 3 |
| Geotrichum spp. | 3 |
| Trichosporon spp. | 3 |
| *Fusarium keratoplasticum* | 1 |
| Pneumocystis jirovecii pneumonia | 1 |
| Unidentified budding yeast cell with pseudohyphae | 13 |
| Serum GM positive patients | 5 |
| Tuberculosis | 5 |
| *Staphylococcus aureus* | 2 |
| Coagulase-negative staphylococci | 4 |
| *Streptococcus pyogenes* | 1 |
| *Streptococcus suis* | 1 |
| *Streptococcus pneumoniae* | 2 |
| *Enterococcus faecium* with unidentified budding yeast cell | 3 |
| *Enterococcus faecalis* | 7 |
| *Escherichia coli* (ESBL producing) | 10 |
| *Klebsiella pneumonia* (ESBL producing) | 8 |
| *Citrobacter freundii* | 2 |
| *Morganella morganii* | 4 |
| *Proteus vulgaris* | 4 |
| *Proteus mirabilis* | 3 |
| *Serratia marcescens* | 4 |
| *Salmonella sp.* | 1 |
| *Acinetobacter baumannii* | 13 |
| *Pseudomonas aeruginosa* | 11 |
| *Stenotrophomonas maltophilia* | 3 |
| Herpes simplex virus | 3 |
| Hepatitis B virus | 7 |
| Hepatitis C virus | 6 |
| Toxoplasmosis | 3 |
| **Total** | **184** |
